# Effects of Small-Sided Games Training versus High-Intensity Interval Training Approaches in Young Basketball Players

**DOI:** 10.3390/ijerph19052931

**Published:** 2022-03-02

**Authors:** Ersan Arslan, Bulent Kilit, Filipe Manuel Clemente, Eugenia Murawska-Ciałowicz, Yusuf Soylu, Mustafa Sogut, Firat Akca, Mine Gokkaya, Ana Filipa Silva

**Affiliations:** 1 Faculty of Sport Sciences, Tokat Gaziosmanpasa University, Tokat 60250, Turkey; ersan.arslan@gop.edu.tr (E.A.); yusuf.soylu@gop.edu.tr (Y.S.); 2 Faculty of Sport Sciences, Tekirdag Namik Kemal University, Tekirdag 59030, Turkey; bkilit@nku.edu.tr; 3 Escola Superior Desporto e Lazer, Instituto Politécnico de Viana do Castelo, Rua Escola Industrial e Comercial de Nun’Álvares, 4900-347 Viana do Castelo, Portugal; anafilsilva@gmail.com; 4 Research Center in Sports Performance, Recreation, Innovation and Technology (SPRINT), 4960-320 Melgaço, Portugal; 5 Instituto de Telecomunicações, Delegação da Covilhã, 1049-001 Lisboa, Portugal; 6 Physiology and Biochemistry Department, University School of Physical Education, 51-612 Wroclaw, Poland; eugenia.murawska-cialowicz@awf.wroc.pl; 7 Department of Physical Education and Sports, Middle East Technical University, Ankara 06800, Turkey; msogut@metu.edu.tr; 8 Faculty of Sport Sciences, Ankara University, Ankara 06560, Turkey; fakca@ankara.edu.tr (F.A.); minegokkaya@ankara.edu.tr (M.G.); 9 The Research Centre in Sports Sciences, Health Sciences and Human Development (CIDESD), 5001-801 Vila Real, Portugal

**Keywords:** interval training, agility, psychophysiological responses, physical enjoyment, perceived exertion

## Abstract

This study aimed to investigate the effects of the 6-week small-sided games training (SSGs) vs. high-intensity interval training (HIIT) on the psychophysiological and performance responses, and technical skills of young basketball players. Thirty-two male players (age: 14.5 ± 0.5 years of age) were randomly divided into SSGs group (*n* = 16) and HIIT group (*n* = 16) training methods thrice per week for 6 weeks. The players in the SSGs group performed two 5–8 min of 2 vs. 2 with 2 min rest periods, while the players in HIIT performed 12–18 min of runs at intensities (90 to 95%) related to the velocity obtained in the 30-15 intermittent fitness test (IFT). Pre-testing and post-testing sessions involved assessments of Yo-Yo Intermittent Recovery Test level 1, 30-15 intermittent fitness test, 5 and 30 m sprint times, vertical jump height, repeated sprint ability, defensive and offensive agility, and technical skills. The SSGs group demonstrated significantly higher agility-based technical responses in terms of the control dribbling and shooting skills (*d* = 1.71 vs. 0.20, *d* = 1.41 vs. 0.35, respectively) compared with the HIIT group. Conversely, the HIIT induced greater improvements in 30 m sprint times (*d* = 3.15 vs. 0.68). These findings provided that SSGs in youth basketball players may allow similar positive physical adaptations to HIIT, with an extra advantage of improving technical skills while improving enjoyability.

## 1. Introduction

The activity demands of basketball depend dominantly on anaerobic and aerobic energy metabolism to perform high levels of performance in repeated high-intensity actions during basketball match-play [1,2]. Anaerobic energy metabolism is responsible for high-intensity activities such as jumping, sprinting, and changes of direction, while aerobic energy metabolism dominates low-intensity activities such as jogging, walking, and standing [3]. Competitive young players cover approximately 5.5–7.5 km (high-intensity activities accounts for approximately 15–23% of the total distance covered [4,5] and also perform 750–1050 activities (each lasting from 1 to 3 s), including change of direction, deceleration, and acceleration [1,6] during a basketball match-play. From a kinematic and technical point of view, young players should have not only well-developed aerobic fitness and anaerobic capacity, but also technical skills to perform these performance indicators under match conditions [7].

Small-sided games (SSGs), also known as skill-based conditioning games or game-based training, are used in the training of players of many sports games, especially in basketball. Earlier studies have documented that SSGs, involving real movement actions and technical awareness, are frequently used training methods to improve functional and sport-specific skills of young players [8,9]. Furthermore, SSGs force players to work intensively and keep constant attention to the game under pressure, force greater concentration of attention and greater physiological demands, as well as provoke greater fatigue [10]. SSGs facilitate the development of technical and tactical skills and give rise to significant improvements in physical and physiological performance [11]. SSGs are widely used by basketball coaches in an attempt to simultaneously develop technical and tactical skills under high physical loads [12].

High-intensity interval training (HIIT), one of the alternatives and popular training methods, is described as the intense and intermittent exercises interspersed with recovery periods. HIIT, provides time-efficiency and better improvement in aerobic capacity in athletes [13,14]. Previous studies have shown that HIIT induced an increase in aerobic and anaerobic performance, cardiopulmonary capacity, and a decrease in fat mass [15]. Delextrat et al. [12] showed that the maximum aerobic performance of young basketball players increased 4.1% after a 6-week HIIT program.

Numerous studies have compared the training effects of HIIT and SSGs programs in team sports, particularly in soccer [8,9,16]; however, few studies have investigated the performance responses and technical activities of young basketball players [12,17]. There is no study in the literature that thoroughly compared the effects of HIIT and SSGs training programs on the psychophysiological, performance responses, and technical skills of young basketball players during the preparation period; therefore, the purpose of this study was to investigate the effects of 6 weeks of SSG and HIIT on the psychophysiological, performance responses, and technical skills of young basketball players. We hypothesized that both training methods would lead to a similar improvement in performance responses without any negative effects.

## 2. Materials and Methods

### 2.1. Study Design

A 2-group, randomized, parallel matched-group, and short-term design were used to compare the effects of SSGs vs. HIIT on the physical performances, and psychophysiological and technical responses in young basketball players. The study was conducted during the preparation period. Thirty-two young male basketball players were randomly divided into two training intervention groups, either in SSGs (*n* = 16) or the HIIT (*n* = 16). The players completed a 30-15 intermittent fitness test (30-15 IFT), 5–30 m sprint test, counter-movement jump (CMJ), CMJ with arm (CMJ_arm_), squat jump (SJ), drop jump (DJ), repeated sprint ability (RSA), t-drill and modified t-drill agility (t-drill_mod_), passing skills (PS), control dribbling (CD), shooting skills (SS), and Yo-Yo Intermittent Recovery Test Level 1 (YYIRT-1). The measurements were performed in the same order in both pre-testing and post-testing sessions. Overall, the study was completed in a total of 8 weeks, consisting of 1 week of pre-testing, 6 weeks of training interventions, and 1 week of the post-testing period. Both training interventions were performed thrice a week. Training sessions were separated by 2 days of rest. During the present study, all players performed the same type of training as well as specific SSGs or the HIIT interventions. Stretching and jogging at various intensity levels together with the integration of basketball-specific drills were performed in a standardized 10 min warm-up section. All tests and training started and ended at similar hours of the day [18].

### 2.2. Subjects

Thirty-two young male basketball players (age: 14.5 ± 0.5 years, height: 179.3 ± 3.7 cm, weight: 70.0 ± 3.6 kg, body fat %13.6 ± 1.3) participated in this study. All players were members of the same young basketball team competing in a regional league. They were accustomed to a training workload of 5 training units per week and had been involved in basketball training and matches for at least 3 years. The players were divided into two groups: the SSGs group (*n* = 16, APHV = 14.0 ± 0.4, age: 14.4 ± 0.5 years, height: 178.3 ± 3.7 cm, weight: 70.2 ± 3.3 kg, body fat %13.8 ± 1.3) and the HIIT group (*n* = 16, APHV = 14.0 ± 0.4, age: 14.6 ± 0.5 years, height: 180.2 ± 3.7 cm, weight: 69.8 ± 3.3 kg, body fat %13.5 ± 1.2) according to their age at peak height velocity (APHV). All players and their parents were informed of the research procedures, requirements, benefits, and risks of the study. The written informed consent forms were obtained from all the subjects and their parents. The study was approved by the local university Ethics Committee (59754796-050.99-2019) and was conducted in accordance with the Declaration of Helsinki.

### 2.3. Testing Procedures

The physical and technical tests lasted 7 days for both pre-testing and post-testing assessments. On the first day, the measurements were performed in the following order: anthropometry, jumping, and sprinting tests. On the third day, the change-of-direction tests and the 30-15 Intermittent Fitness test were performed. On the fifth day, technical skill tests and repeated sprint ability tests were performed, respectively. On the seventh day, Yo-Yo Intermittent Recovery Level 1 tests were performed. Five minutes of rest were provided between tests. A standardized warm-up protocol consisting of jogging and dynamic and static stretching was provided before performance tests.

#### 2.3.1. Anthropometric Measurements and Maturity

Body weights were measured using the bioelectrical impedance analyzer (BC-418, Tanita, Tokyo, Japan). The sitting and standing height were measured with a stadiometer (Holtain Ltd., UK). A non-invasive technique proposed by Mirwald et al. [19] was used to estimate the maturity status of the players. Firstly, maturity offset the number of predicted years before or after the APHV, was calculated according to the following equation:

Maturity Offset= −9.236 + 0.0002708 (leg length × sitting height) − 0.001663 (age × leg length) + 0.007216 (age × sitting height) + 0.02292 (weight/height × 100). Then, the maturity offset was subtracted from the chronological age of the players to estimate the APHV.

#### 2.3.2. Change-of-Direction Tests

The t-drill and t-drill_mod_ were applied for the measurement of change-of-direction (COD) performances. These tests are reliable and valid tests for the evaluation of change of direction performance [20], which were performed on an indoor basketball court. According to the procedures suggested by Pauole et al. [21] in the t-drill test, including basketball-specific movements such as sprinting, shuffling, and backpedaling, each player covered a total distance of 36.56 m. The same test protocol was also used in t-drill_mod_ test except for the total covered distance. All players performed each COD test with 3 min of passive resting between consecutive trials and 5 min between COD tests to minimize physical fatigue and risk of injury. Times in COD tests were measured using photocells (Newtest Powertimer 300-series, Newtest Oy, Tyrnävä, Finland). The ICC was 0.95 and 0.93 in t-drill and t-drill_mod,_ respectively.

#### 2.3.3. Jumping Tests

The vertical jump heights were assessed by CMJ, CMJarm, SJ, and DJ for each player using a portable force plate (Newtest Powertimer 300-series, Newtest Oy, Tyrnävä, Finland). While the CMJ was performed with hands placed on the hips to minimize the contribution of the upper limbs, the players performed the CMJarm with free arm swing [22]. The SJ was performed starting from a static semi squatting position maintained for a second and without any preliminary movement. The DJ was performed starting from a standing position on a 30 cm height, dipping, and then extending the knee in a continuous movement [23]. The players performed each jumping test with 2 min of passive resting between consecutive trials and 5 min between jumping tests. The ICC was 0.96, 0.94, 0.95, and 0.94 in CMJ, CMJarm, SJ, and DJ respectively.

#### 2.3.4. Sprinting and Repeated Sprint Tests

Each player stood ~70 cm behind the start line and then performed a 30 m sprinting test (with 5–10 and 20 m splits). The player performed three trials separated by 2 min of passive resting for this test. The RSA test involved 6 repetitions of maximal 2 × 15 m shuttle sprints (~6 s) departing every 20 s [24]. The player performed starting from a split-stance standing and sprinted for 15 m, changed the direction at 180°, and sprinted back to the finish line. The players were asked to alternate legs between sprints to minimize imbalances between lower limbs and the risk of injury. Times in these tests were measured using photocells (Newtest Powertimer 300-series, Newtest Oy, Tyrnävä, Finland). The ICC was 0.96 and 0.94 in the 30 m sprinting test and RSA, respectively.

#### 2.3.5. Technical Skill Tests

The technical skills were assessed by CD, PS, and SS in the present study. The CD consists of running with ball-handling skills. Each player started with dribbling and covered a total distance of 17.9 m while passing the cones and changing hands [25]. In the PS test, the players were instructed to perform 2-handed chest passes behind a line placed 2.45 m from the first and sixth target on the wall, while side-shuffling during the 30 s. Passes were executed in the following sequence order: A-B-C-D-E-F-F-E-D-C-B-A-A-B-… during the test. Two points were given when the ball hit the target or targets’ border, while the players were awarded one point when the ball touched the spaces between targets [26]. In the SS test, the players were instructed to shoot, starting behind any of the 5 different positions approximately 4.54 m from the basket, following getting their rebound and immediately dribbling to another position during the 60 s. They were also instructed to attempt at least 1 shot from each position. The players were allowed to attempt a maximum of 4 lay-ups during each trial, but these could not be performed in succession. In total, 2 and 1 points were given for each successful and unsuccessful shot, respectively [26]. The ICC was 0.93, 0.95, and 0.94 in CD, PS, and SS, respectively.

#### 2.3.6. 30-15 Intermittent Fitness Test

The 30-15 IFT, which consists of a 30-s run and 15-s passive rest on a standardized indoor basketball court, is an acoustically progressive field test. Each player performed the 30-15 IFT to determine players’ individual high-intensity intermittent running performance with changes of direction. The players were instructed to complete as many stages as possible during the test. The test ended when a player could no longer maintain the imposed running speed or when a player was unable to reach a 3 m zone around each line at the moment of the audio signal 3 times consecutively. The running speed during the last successfully completed stage was recorded as velocity obtained in the intermittent fitness test (VIFT) [27].

#### 2.3.7. Yo-Yo Intermittent Recovery Level 1 Test

To evaluate aerobic capacity, the YYIRT-1 was performed according to procedures described by Bangsbo et al. [28]. Briefly, the test consisted of 20 m shuttle runs with an increasing velocity during the test. After the test, the estimated VO_2max_ was calculated using the following formula

VO_2max_ = 36.4 + (0.0084 × covered distance in YYIRT-1)

Except for YYIRT-1, 30-15 IFT, and RSA tests, two trials were given, and the better score was used for statistical analysis. All players were familiar with all tests used in this study and were verbally encouraged by their team coach to exert maximal efforts during the testing and training sessions. All tests and training were performed on an indoor basketball court with a sprung wooden floor at a similar time of the day for similar chronobiological characteristics [18].

### 2.4. Training Interventions

The 6-week study was conducted during the preparation period. Each daily training session started with a 10-min standardized warm-up consisting of jogging, dynamic stretching, and sprinting with the integration of basketball-specific technical actions. Following the warm-up, the players performed either SSGs or a HIIT session with the same amount of total training time during each training session. To minimize the adverse effects of fatigue on psychological, physiological, and performance responses, each training intervention was performed by at least 2 days interval. The players performed intermittent running at 90–95% of players’ VIFT or 15 s, followed by 15 s of passive resting in the HIIT sessions around a standard athletics track. Meanwhile, players in SSGs performed full-court 2-a-side SSGs (one of the most popular basketball-specific game strategies), lasting for 10 and 18 min per training session, with 2 min passive resting between bouts of 2-a-side SSGs, were also performed because the players showed similar exercise intensity (>85% of their individual HR_max_) to the running-based HIIT exercises in young basketball players [29,30]. Each team in SSGs was selected by their coach to avoid having unbalanced groups, especially in regard to physical fitness and technical abilities, and these teams remained stable during the study. The rating of perceived exertion (RPE) was obtained using the category ratio scale (CR-10) to calculate internal training load (ITL) immediately after the completion of each session [31]. The scale was introduced at the beginning to familiarize the players. All players also completed the short form of the physical activity enjoyment scale (PACES). The scale includes 5 items scored on a 1–7 Likert scale and has been validated as a marker of enjoyment level in activity by Turkish youth [32].

### 2.5. Statistical Analyses

Data were represented as mean ± SD. Group differences in psychophysiological responses in terms of RPE, PACES, RSME, and also ITL between pre- and post-test results were assessed using the independent sample t-test. The intraclass correlation coefficient (ICC) was used to determine the test–retest reliability of the performance tests. A 2-way repeated-measures analysis of variance was used to assess the effects of group (SSGs vs. HIIT) and time (pre vs. post). Effect sizes (Cohen’s d) were also calculated for each dependent variable. Cohen’s *d* were considered trivial (<0.20), small (0.20–0.59), moderate (0.6–1.19), large (1.2–1.99), and very large (≥2.0) [33]. All statistical analyses were computed using SPSS version 24.0 for Windows (SPSS Inc., Chicago, IL, USA). Statistical significance was set at the level of *p* ≤ 0.05.

## 3. Results

Table 1 shows the weekly psychophysiological responses in terms of RPE, PACES, RSME, and ITL. The RPE responses to HIIT sessions were significantly higher than those to SSGs sessions (8.8 ± 0.4 vs. 7.7 ± 0.3; *p* ≤ 0.05, *d* = 3.27 (very large effect)). The HIIT showed a higher RSME responses than those to SSGs sessions (97.9 ± 8.6 vs. 63.2 ± 5.8; *p* ≤ 0.05, *d* = 4.73 (very large effect)). Furthermore, The HIIT also demonstrated a higher ITL responses than those to SSGs sessions (457.4 ± 19.4 vs. 400.8 ± 14.6; *p* ≤ 0.05, *d* = 3.29 (very large effect)). Conversely, the PACES scores from the SSGs were significantly higher than those to HIIT sessions (28.8 ± 1.8 vs. 22.9 ± 1.4; *p* ≤ 0.05, *d* = 3.67 (very large effect)).

**Figure 1:**
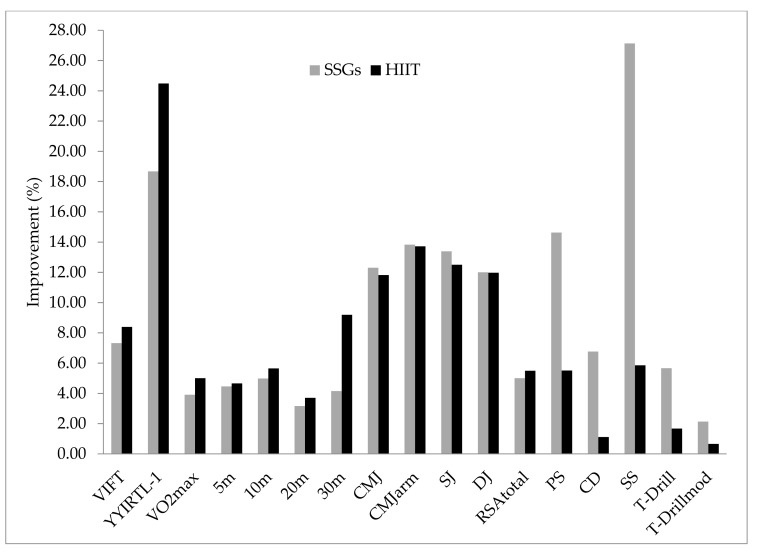
Improvement in performance and technical responses following the SSGs and HIIT interventions.

In terms of within-group comparisons, significant changes were observed in all measured performance responses (*p* ≤ 0.05, *d* values ranging from trivial to very large effects) (Table 2). Moreover, the between-group comparison showed that SSGs demonstrated significantly higher performance responses than the HIIT group in the SD and SS test performances (*p* ≤ 0.05, *d* = ranging from 1.41 to 1.71 (large effects)). Conversely, the HIIT group demonstrated significantly higher performance responses in the 30-m sprinting time (*p* ≤ 0.05, *d* = 0.68 (very large effects)) (Figure 1).

## 4. Discussion

The novelty of the current research was the comparison between SSG vs. HIIT training on the physical fitness adaptations (e.g., locomotor profile, jumping ability, sprint, and change-of-direction) and technical abilities of basketball players. Such kind of analysis is scarce in basketball, and this provides extra information for coaches and practitioners. Main findings revealed that both SSG and HIIT training were effective in improving locomotor profile, aerobic fitness, repeated sprint ability, linear speed between 5- and 20-m, vertical jump, and T-drill significantly and similarly. Although both had a significant positive effect on technical skills, the SSG group performed significantly better than the HIIT group. In comparison to SSG, however, the HIIT group benefited significantly in 30 m.

SSGs are typically used in small doses to improve athletes’ aerobic power because they tax both anaerobic and aerobic systems in short bursts of activity [29]. Similarly, with short HIIT, SSGS improves maximal oxygen uptake by focusing on maximal cardiac outputs [34], while also allowing active glycolytic [17] and neuromuscular systems [12]. As a result, it is reasonable to expect (if well designed) that SSGs will improve energetic systems, thereby providing support for improving locomotor profile, aerobic fitness, or repeated-sprint ability. In the current study, both groups (SSG and short-HIIT) significantly improved final velocity at 30-15 IFT, aerobic capacity in the YYIRT-1, and RSA tests. These study results are in line with similar study designs performed in youth soccer players [8] and youth basketball players [35].

Knowing that aerobic fitness may significantly help RSA (primarily the outcomes related to average or total, because with the progression of repetitions, aerobic fitness plays a more important role) [36], it is reasonable to speculate that SSGs may indirectly benefit RSA by assisting aerobic fitness and/or the ability to recover faster between intermittent actions [37]. Similarly, aerobic fitness may influence the 30-15 IFT, but not exclusively. Because the 30-15 IFT is a multi-dependent test influenced by aerobic power, change-of-direction, or neuromuscular readiness [38], SSGs and short HIIT may have provided additional support with improvements in change-of-direction or neuromuscular readiness. SSGs, in particular, expose players to a high frequency of direction changes, accelerations, and decelerations (neuromuscular stimulus) [39], which may indirectly benefit the ultimate performance at 30-15 IFT. This is supported by the fact that vertical jump in counter-movement and drop jumps have also improved, potentially competing to justify the improvements in 30-15 IFT [40].

It was also interesting to note that, with the exception of the longer test, both training interventions resulted in similar significant improvements in almost all linear speed tests. SSGs are typically played in small longitudinal sizes, so the lack of space to reach maximal speed in distances associated with maximal velocity can be affected or not as well developed as in short HIIT, which can cover longer distances (considering the linear space) [10,41]. Furthermore, the acceleration period is associated with shorter than 30 m, which is more related to the type of SSGs stimulus performed in small spaces [42]. In terms of technical dimensions, while both groups improved their skills, those who participated in SSGs benefited significantly more in control dribbling and shooting. This is related to the fact that SSG is a drill-based exercise in which technical events emerge from the game’s dynamic [43]. As a result, the continuous game stimulus may provide additional support for players’ technical abilities without jeopardizing positive physical adaptations. Our findings are also consistent with previous research comparing SSGs and HIIT in youth basketball players [12].

Surprisingly, the monitoring process for intensity levels experienced by players during training sessions and enjoyment revealed that SSGs provided greater enjoyability, less intensity perception, and less mental effort perception, which is consistent with previous reports [9,44]. This evidence is extremely important because SSGs in youth basketball players may allow almost identical positive physical adaptations as HIIT, with the added benefit of improving technical skills while improving enjoyment and adherence (eventually).

There are some limitations to the current research. The specific age group may aid in favoring adaptations. More research in higher competitive levels and ages may be warranted. Furthermore, generalizability cannot be performed because the study sample is small, and the sample was chosen for convenience and the fact that it is the result of a single team analysis. Furthermore, no control group that did anything other than HIIT-based regimens (in which SSGs were included) or did not do a specific intervention was tested. As a result, future studies should involve more teams, including a wider range of age groups, consider the effect of baseline levels, and compare with different non-intervention controls.

## 5. Conclusions

This study revealed that SSG and HIIT-based training interventions are both effective for improving the general physical fitness and technical abilities of basketball players. In specific, post-training analysis revealed that both SSGs and HIIT are considered effective for improving youth basketball players’ locomotor profile, aerobic fitness, speed, repeated sprint ability, vertical jump, and technical skills. Considering these results, the coaches are free to choose the most appropriate and adequate training method, since both provide similar effects in physical fitness and technical abilities.

## Figures and Tables

**Table 1:** Description of the 6 weeks of SSGs and HIIT training programs, the weekly psychophysiological responses, and internal training load.

Weeks	SSGs	RPE	PACES	RSME	ITL	HIIT	RPE	PACES	RSME	ITL
1	2 × (2 × 2.30 min), 2 min rest	7.9 ± 0.6	27.9 ± 2.5	85.8 ± 9.8	285.3 ± 22.3	2 × (6 min of 15″–15″ at 90% of VIFT)	8.8 ± 0.5	21.2 ± 2.5	107.3 ± 14.4	300.4 ± 20.9
2	2 × (2 × 3 min), 2 min rest	7.8 ± 0.7	26.9 ± 2.7	75.2 ± 6.9	328.1 ± 27.5	2 × (7 min of 15″–15″ at 90% of VIFT)	8.7 ± 0.7	22.2 ± 1.9	110.8 ± 11.5	347.8 ± 28.1
3	2 × (2 × 3.30 min), 2 min rest	7.7 ± 0.6	28.3 ± 2.8	66.9 ± 9.9	372.0 ± 27.7	2 × (8 min of 15″–15″ at 90% of VIFT)	8.7 ± 0.7	22.6 ± 1.6	107.7 ± 9.6	396.0 ± 30.2
4	2 × (2 × 3 min), 2 min rest	8.0 ± 0.5	29.2 ± 2.9	58.1 ± 9.2	432.0 ± 27.8	2 × (7 min of 15″–15″ at 95% of VIFT)	8.8 ± 0.8	22.5 ± 1.5	96.7 ± 6.8	452.2 ± 34.8
5	2 × (2 × 3.30 min), 2 min rest	7.6 ± 0.5	29.2 ± 2.5	49.2 ± 8.7	453.7 ± 30.7	2 × (8 min of 15″–15″ at 95% of VIFT)	8.5 ± 0.6	24.4 ± 1.4	87.7 ± 6.4	481.8 ± 34.3
6	2 × (2 × 4 min), 2 min rest	7.4 ± 0.5	31.8 ± 1.5	43.9 ± 6.1	535.5 ± 36.9	2 × (9 min of 15″–15″ at 95% of VIFT)	9.1 ± 0.7	24.3 ± 1.3	77.1 ± 8.1	596.2 ± 44.3

SSGs: small-sided games training; HIIT: high-intensity interval training; RPE: rating of perceived exertion; PACES: physical activity enjoyment scale; RSME: rating scale mental effort; ITL: internal training load; VIFT: Maximum speed reached in the last stage of the 30-15 Intermittent Fitness Test.

**Table 2:** Effect of both training methods on performance responses of the participants.

		SSGs				HIIT		
	Pre	Post	d	Descriptor	Pre	Post	d	Descriptor
V_IFT_ (km.h^−1^)	13.31 ± 0.63	14.28 ± 0.58 *	1.60	Large	13.66 ± 0.77	14.78 ± 0.52 *	1.70	Large
YYIRTL-1 (m)	1177.50 ± 122.17	1392.50 ± 103.51 *	1.90	Large	1130.00 ± 100.66	1402.50 ± 79.29 *	3.01	Very Large
VO_2max_ (mL.min^−1^.kg^−1^)	46.29 ± 1.03	48.10 ± 0.87 *	1.90	Large	45.89 ± 0.85	48.18 ± 0.67 *	2.99	Very Large
5 m (s)	1.16 ± 0.05	1.11 ± 0.04 *	1.10	Moderate	1.14 ± 0.07	1.09 ± 0.06 *	0.77	Moderate
10 m (s)	1.96 ± 0.19	1.87 ± 0.18 *	0.49	Small	1.93 ± 0.13	1.82 ± 0.13 *	0.85	Moderate
20 m (s)	3.47 ± 0.24	3.36 ± 0.22 *	0.48	Small	3.43 ± 0.21	3.30 ± 0.19 *	0.65	Moderate
30 m (s)	5.41 ± 0.34	5.18 ± 0.34 *	0.68	Moderate	5.35 ± 0.17	4.86 ± 0.14 *Ω	3.15	Very Large
CMJ (cm)	31.55 ± 2.13	35.40 ± 1.83 *	1.94	Large	31.23 ± 1.62	34.91 ± 1.77 *	2.17	Very Large
CMJ_arm_ (cm)	35.38 ± 2.31	40.27 ± 2.56 *	2.01	Very Large	34.92 ± 1.77	39.71 ± 2.07 *	2.49	Very Large
SJ (cm)	29.66 ± 2.35	33.60 ± 2.32 *	1.69	Large	29.46 ± 1.85	33.12 ± 1.80 *	2.00	Large
DJ (cm)	34.77 ± 3.24	38.92 ± 3.49 *	1.23	Large	34.20 ± 2.26	38.29 ± 2.57 *	1.69	Large
RSA_total_ (s)	36.87 ± 1.29	35.03 ± 1.30 *	1.42	Large	36.66 ± 0.83	34.65 ± 0.79 *	2.48	Very Large
PS (points)	53.25 ± 4.49	60.88 ± 3.26 *	1.94	Large	52.50 ± 7.64	55.25 ± 7.26 *	0.37	Small
CD (s)	10.68 ± 0.49	9.95 ± 0.35 *Ω	1.71	Large	10.75 ± 0.61	10.63 ± 0.56 *	0.20	Trivial
SS (s)	21.50 ± 4.23	26.88 ± 3.34 *Ω	1.41	Large	21.00 ± 3.27	22.13 ± 3.14 *	0.35	Small
T-Drill (s)	12.56 ± 0.85	11.84 ± 0.73 *	0.91	Moderate	12.46 ± 0.62	12.26 ± 0.61 *	0.32	Small
T-Drill_mod_ (s)	6.64 ± 0.39	6.50 ± 0.37 *	0.39	Small	6.65 ± 0.54	6.60 ± 0.55 *	0.09	Trivial

Data are Mean ± SD. V_IFT_: velocity reached at the end of the 30-15 IFT test: YYIRTL-1: Yo-Yo Intermittent Recovery Test level 1; VO_2max_: maximal oxygen uptake; CMJ: counter-movement jump; CMJ_arm_: counter-movement jump with arms; SJ: squat jump; DJ: drop jump; RSA_total_: total time during repeated sprint ability test; PS: Passing Skills; CD: Control Dribbling; SS: Shooting Skills; T-Drill_mod_: Modified T-Drill. * Significant difference between pre- and post-training. Ω Significant difference between groups.

## Data Availability

Not applicable.
